# 35/w – gelegentliches sensorisches Missempfinden im Bein und Schmerzen im Rücken: Vorbereitung auf die Facharztprüfung: Fall 51

**DOI:** 10.1007/s00132-020-04045-w

**Published:** 2020-12-11

**Authors:** Daniel Sauer

**Affiliations:** 1 Wirbelsäulenzentrum, Schön Klinik München Harlaching, Harlachinger Str. 51, 81547 München, Deutschland; 2 Paracelsus Medical University, Salzburg, Österreich

**Keywords:** Konservative Versorgung, Konus-Kauda-Syndrom, Nervenwurzelkompression, Operative Versorgung, Paresen

## Prüfungssimulation

### Fallschilderung

Die 35-jährige Frau K. kommt in Ihre Sprechstunde und berichtet über seit längerem bestehende Schmerzen im Rücken und der unteren Extremität. Sie habe die Schmerzen im unteren Rücken mit gelegentlichen Gefühlsstörungen im Bein ohne ein erinnerliches Trauma bekommen.

## Prüfungsfragen

- Welche weiteren anamnestischen Fragen bzw. klinischen Untersuchungen interessieren Sie noch im Besonderen?
- Welche weiterführende Diagnostik halten Sie für notwendig? Welche Differenzialdiagnosen haben Sie in Betracht gezogenen und warum? Wie können Sie diese ausschließen?
- Was wissen Sie über das Krankheitsbild hinsichtlich des Entstehungsmechanismus und der Diagnosekriterien?
- Was sind die gängigen radiologischen Klassifikationssysteme?
- Wie kann die Prognose des Befundes/der Erkrankung abgeschätzt werden?
- Was sind die Kriterien für eine konservative oder operative Behandlung?
- Welches weitere Vorgehen besprechen Sie in diesem Fall mit Frau K.?
- Was sind die Vor- und Nachteile der endoskopischen operativen Versorgung?

### Antworten

#### Welche weiteren anamnestischen Fragen bzw. klinischen Untersuchungen interessieren Sie noch im Besonderen?

##### Anamnese.

Sind die Schmerzen akut oder schleichend eingetreten? Können Sie den Charakter der Schmerzen beschreiben? Wandern die Schmerzen und Beschwerden in der Ausdehnung und Erscheinung? Besteht eine aktuelle Infektion (Krankheitsgefühl, Fieber)? Bestehen Vorerkrankungen? Sind eine Blasen- oder Mastdarmstörung bekannt?

##### Der Fall.

Die Schmerzen seien akut ohne ein erinnerliches Trauma über Nacht eingetreten. Zunächst seien die Schmerzen auf den unteren Rücken mit diffusem und dumpfem Schmerzcharakter beschränkt gewesen. Nun wechselt der Schmerz seine Position mit „vom Rücken ins Bein ziehend“. Vorerkrankungen sind keine bekannt.

##### Klinische Untersuchung.

Gezielt sollte nach spezifischen Befunden gesucht werden.

- Untersuchungsgang: Inspektion/Palpation/Funktionsprüfung
- Zeigt sich eine palpable Muskelverhärtung?
- Besteht eine Einschränkung der Beweglichkeit des Beines/der unteren Extremität (Lasègue-Test)?
- Ist eine Blasen- oder Mastdarmstörung vorhanden?
- Motorische und sensorische funktionelle Untersuchung der Dermatome der unteren Extremität.

##### Der Fall.

Geringe palpable paravertebrale Muskelverspannung, leicht links hinkendes Gangbild. Es zeigt sich keine myofasziale Dysfunktion am Iliosakralgelenk, Hüfte, Knie oder oberen Sprunggelenk. Positiver Lasègue-Test links bei 20 °*, *geringer Kraftverlust des Fußsenkers links, keine Blasen- oder Mastdarmstörung, geringe Hypästhesie an der Ferse links, ansonsten kein sensomotorisches Defizit.

#### Welche weiterführende Diagnostik halten Sie für notwendig? Welche Differenzialdiagnosen haben Sie in Betracht gezogenen und warum? Wie können Sie diese ausschließen?

- Bildgebung:
  - Röntgen der LWS in 2 Ebenen (Augenmerk auf: Alignement, Anzahl der Wirbelkörper lumbal, Gleitwirbel, Fraktur)
  - MRT der LWS: essenziell!
  - CT wenn kein MRT möglich
- Laboruntersuchung (Leukozyten, C‑reaktives Protein) bei V. a. Infektionsgeschehen
- EMG

##### Der Fall.

**Figure 1:**
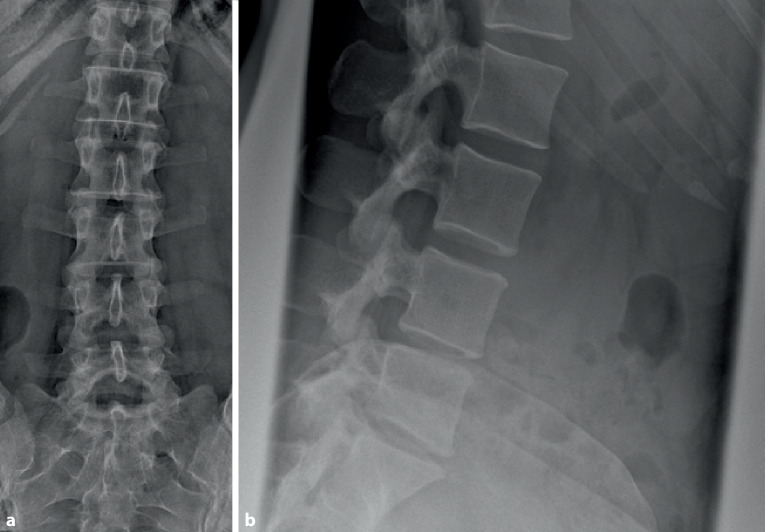

Die Labordiagnostik zeigt einen unauffälligen Befund ohne laborchemische pathologische Infektionsparameter, die Röntgenaufnahme zeigt eine geringe Bandscheibenhöhenminderung ohne Anzeichen eines Gleitwirbels, leichte kyphotische segmentale Stellung (Abb. 1 und 2).

**Figure 2:**
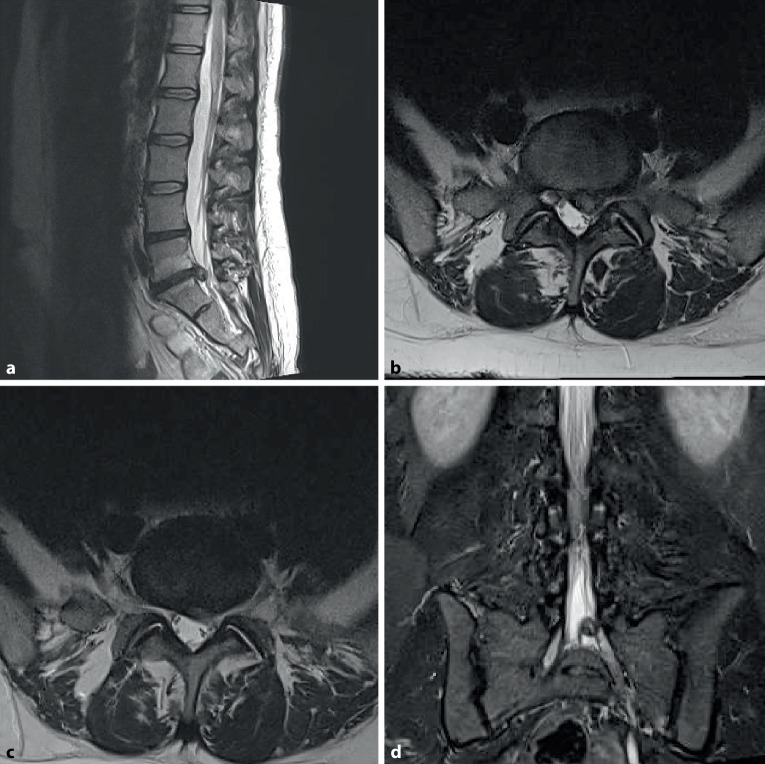

##### Differenzialdiagnosen.

- Piriformis-Syndrom durch klinische Untersuchung und MRT-Diagnostik der LWS auszuschließen.
- Periphere Läsion des beteiligten Nerven: Durch klinische Untersuchung und Anamnese auszuschließen, i. d. R. keinerlei Rückenschmerz vorhanden. Meistens schmerzlose Parese.
- periphere arterielle Verschlusskrankheit (meistens ältere Patienten, Raucher deutlich erhöhte Inzidenz, Beschwerdezunahme beim Gehen)
- Arthrose oder Impingement der Hüftgelenke durch klinische Untersuchung und Anamnese auszuschließen.
- Bannwarth-Syndrom, im Rahmen einer Lyme-Borreliose auftretend, schmerzhafter Entzündung von peripheren Nerven.
- Achillessehnenriss: Durch klinische Untersuchung meist einfach zu erkennen (Sehnenlücke).

##### Der Fall.

Es handelt sich um einen lumbalen Bandscheibenvorfall bei Bandscheibendegeneration mit einer radikulären Symptomatik.

#### Was wissen Sie über das Krankheitsbild hinsichtlich des Entstehungsmechanismus und der Diagnosekriterien?

##### Multifaktorielle Ursache.

- Umstrukturierung der Kollagenfasern, mechanische Belastung, Abnahme der Proteoglykankonzentration
- Genetische Faktoren: Verwandte ersten Grades zeigen ein erhöhtes Erkrankungsrisiko.
- Traumatische Bandscheibenvorfälle sind sehr selten und auf wiederholte Extrembelastungen im Sinne von Hyperflexion/Rotationsmechanismen zurückzuführen [3].

##### Diagnose.

- Typische Anamnese und klinische Untersuchung, Verschlechterung der Schmerzen beim Husten, Niesen und Valsalva-Manöver
- MRT: Goldstandard zum Nachweis eines Bandscheibenvorfalles und Lagebezug zur Nervenwurzel, Ausschluss einer neuralen Kompression

#### Was sind die gängigen radiologischen Klassifikationssysteme?

**Figure 3:**
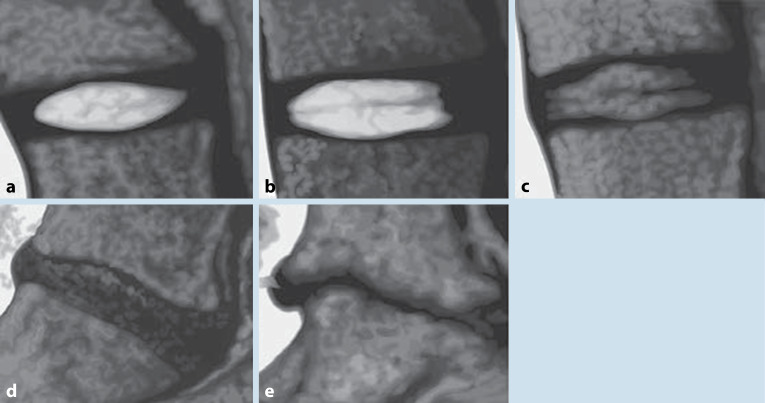

In Abb. 3 und Tab. 1 sind die gängigen Klassifikationssysteme dargestellt.

**Figure 4:**
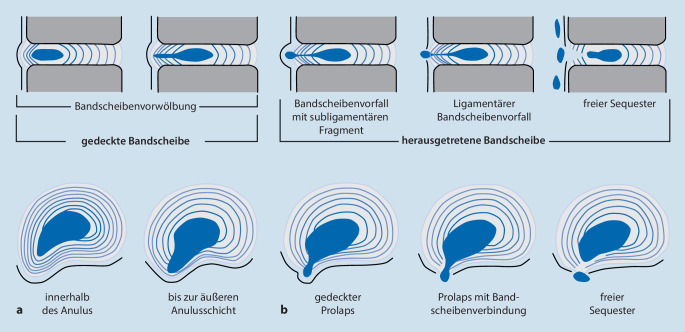

- Höhe: diskal, infradiskal, supradiskal
- Lokalisation: dorsomedial, dorsolateral, intraforaminal, extraforaminal (Abb. 4) Klassifikation des Bandscheibenvorfalles nach Kramer et al. [2]

**Table 1:** 

Einteilung der Bandscheibendegeneration	Struktur der Bandscheibe	Abgrenzung von Anulus und Nucleus	MRT-Signalintensität	Bandscheibenraumhöhe
Grad I	Homogen, weiß	Klar	Hyperintens, isointens zu Liquor	Normal
Grad II	Inhomogen mit/ohne horizontale Banden	Klar	Hyperintens, isointens zu Liquor	Normal
Grad III	Inhomogen, grau	Unklar	Intermediate	Normal bis gering verringert
Grad IV	Inhomogen, grau oder schwarz	Nicht möglich	Intermediate bis hypointens	Normal bis ausgeprägt verringert
Grad V	Inhomogen schwarz	Nicht möglich	Hypointens	Kollabiert

#### Wie kann die Prognose des Befundes/der Erkrankung abgeschätzt werden?

Anhand des klinischen, körperlichen Untersuchungsbefundes mit der Anamnese und Erhebung des neurologischen Status. Beurteilung der aktuellen MRT-Diagnostik:

- Lokalisation und Ausdehnung des Bandscheibenvorfalles: median, mediolateral, lateral/rezessal, intraforaminal, extraforaminal, kaudal/kranial sequestriert
- frisch/alt, weich/harter Vorfall; Knorpelgewebe der Endplatte
- Ausmaß der Nervenkompression; Konus-Kauda-Symptomatik

##### Der Fall.

Bei einem mediolateral liegenden Bandscheibenvorfall mit Kompression der Nervenwurzel ohne Konus-Kauda-Symptomatik bei kurzzeitiger Beschwerdesymptomatik mit einem geringen Kraftverlust des Kennmuskels von 4/5 nach Janda ist eine konservative Therapie durchaus vertretbar.

#### Was sind die Kriterien für eine konservative oder operative Behandlung?

##### Konservativ.

Es kann davon ausgegangen werden, dass ca. 80 % der betroffenen Patienten eine Besserung der Beschwerden durch die konservative Therapie erfahren werden.

- Bettruhe nur in der Akutphase bei Immobilisation.
- Frühe Mobilisierung mit leichter bis mäßiger Belastung erstrebenswert.
- Frühzeitige und ausreichende analgetische Therapie mit nichtsteroidalen Antirheumatika (NSAR) und ggf. Muskelrelaxanzien, bei starken Schmerzen können kurzfristig retardierte Opioide indiziert sein.
- Bei Entwicklung chronischer Schmerzen ist eine multimodale Schmerztherapie (Kombination interdisziplinärer Fachgruppen wie z. B. Sporttherapeuten, Physiotherapie und psychologische Psychotherapeuten [Verhaltenstherapie, Schmerzbewältigungsprogramme]) indiziert.

##### Operativ.

- Bei unter konservativer Therapie persistierenden Beschwerden.
- Beim Vorliegen einer Konus-Kauda-Symptomatik besteht eine absolute Notfallindikation.
- Rasch progrediente Parese und schwere neurologische Ausfallerscheinungen 0–2/5 Kraftgrad nach Janda.
- Ausgeprägte Schmerzen unter forcierter Schmerztherapie.

#### Welches weitere Vorgehen besprechen Sie in diesem Fall mit Frau K.?

##### Der Fall.

Bei Frau K. ist zunächst eine konservative Therapie mittels oraler Schmerztherapie und Physiotherapie indiziert/empfehlenswert. Bei stärkeren Schmerzen ist die selektive Infiltration unter Bilddokumentation gerechtfertigt, ggf. mit zusätzlicher oraler Steroidtherapie. Konsequenter Muskelaufbau der rumpfstabilisierenden Muskulatur.

#### Was sind die Vor- und Nachteile der endoskopischen operativen Versorgung?

Bei der aktuellen operativen Versorgung der Patienten ist der Goldstandard die mikrochirurgische Operationstechnik. In den letzten Jahren hat sich ein nahezu gleichwertiges etabliertes operatives Verfahren entwickelt: Die endoskopische Versorgung des Bandscheibenvorfalls.

##### Vorteile.

- kleine Hautinzision 5–7 mm
- atraumatischer Zugang
- antiinflammatorischer Effekt bei permanenter Lavage
- kurze Operationsdauer
- Same-Day-Surgery möglich
- geringer perioperativer Schmerz
- gute klinische Ergebnisse

##### Nachteile.

- technisch aufwendiges Operationsverfahren
- längere und flache Lernkurve
- gegebenenfalls Konversion (bei Komplikationen) auf das mikrochirurgische Operationsverfahren nötig
